# Autoimmune pulmonary alveolar proteinosis prior to myelodysplastic syndrome

**DOI:** 10.1002/rcr2.569

**Published:** 2020-05-05

**Authors:** Chuan Tai Foo, Louis Chhor, Francis Thien

**Affiliations:** ^1^ Eastern Health Melbourne VIC Australia; ^2^ Eastern Health Clinical School Monash University Melbourne VIC Australia

**Keywords:** Autoimmune, myelodysplastic syndrome, pulmonary alveolar proteinosis

## Abstract

We report the first case of autoimmune pulmonary alveolar proteinosis (PAP) associated with and preceding myelodysplastic syndrome. A 74‐year‐old female with a history of polymyalgia rheumatica presented with six months history of progressive exertional breathlessness. Examination revealed bilateral chest crackles and exertional desaturation. A diagnosis of autoimmune PAP was made based on the presence of autoantibodies to granulocyte‐macrophage colony‐stimulating factor and characteristic findings on chest computed tomography, bronchoalveolar lavage, and transbronchial biopsies. Bilateral whole lung lavage was performed with prompt improvement in symptoms. Fourteen months later, she presented with new breathlessness and was diagnosed with myelodysplasia on bone marrow biopsy. No recurrence of alveolar proteinosis was detected. This case highlights the importance of follow‐up and screening of patients with autoimmune PAP for haematological conditions.

## Graphical abstract

We report the first case of autoimmune pulmonary alveolar proteinosis associated with and preceding myelodysplastic syndrome.

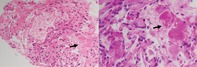

## Introduction

Pulmonary alveolar proteinosis (PAP) is a rare disorder characterized by accumulation of lipoproteinaceous material within the alveolar space. Clinical presentation varies from asymptomatic to respiratory failure. Autoimmune PAP is defined by the presence of autoantibodies to granulocyte‐macrophage colony‐stimulating factor (GM‐CSF) and, unlike secondary PAP, has not been reported to be associated with haematological malignancies. We present the first case of autoimmune PAP associated with and preceding myelodysplastic syndrome (MDS).

## Case Report

A 74‐year‐old female was referred to hospital by her general practitioner with a chest X‐ray showing bilateral widespread reticular changes. The X‐ray was performed to investigate six months of progressive shortness of breath associated with a non‐productive cough and a significant reduction in her usual exercise capacity.

On history, the patient is a lifelong non‐smoker and had no infective or cardiac symptoms. No occupational or environmental exposures were identified. Relevant past medical history includes polymyalgia rheumatica (PMR) managed with sulfasalazine for the past six years.

On examination, the patient was not dyspnoeic and had a room air peripheral capillary oxygen saturation (SpO_2_) of 97% at rest. SpO_2_ decreased to 84% on 50 m exertion. Chest auscultation revealed sparse bilateral crackles. Other vitals and the remainder of the clinical examination were normal.

**Figure 1:**
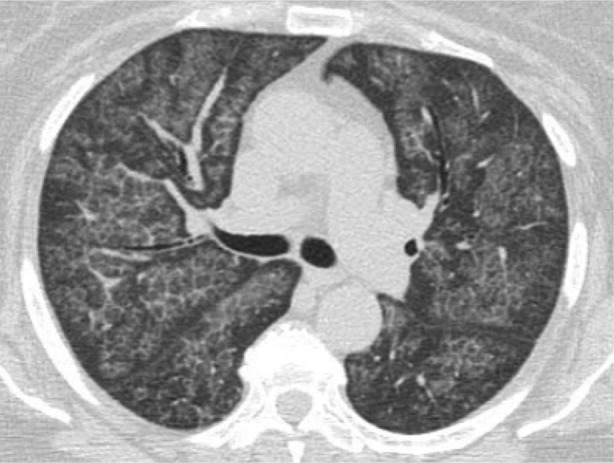
Axial chest high‐resolution computed tomography (HRCT) showing bilateral widespread ground‐glass opacification with superimposed septal thickening referred to as “crazy‐paving” appearance.

**Figure 2:**
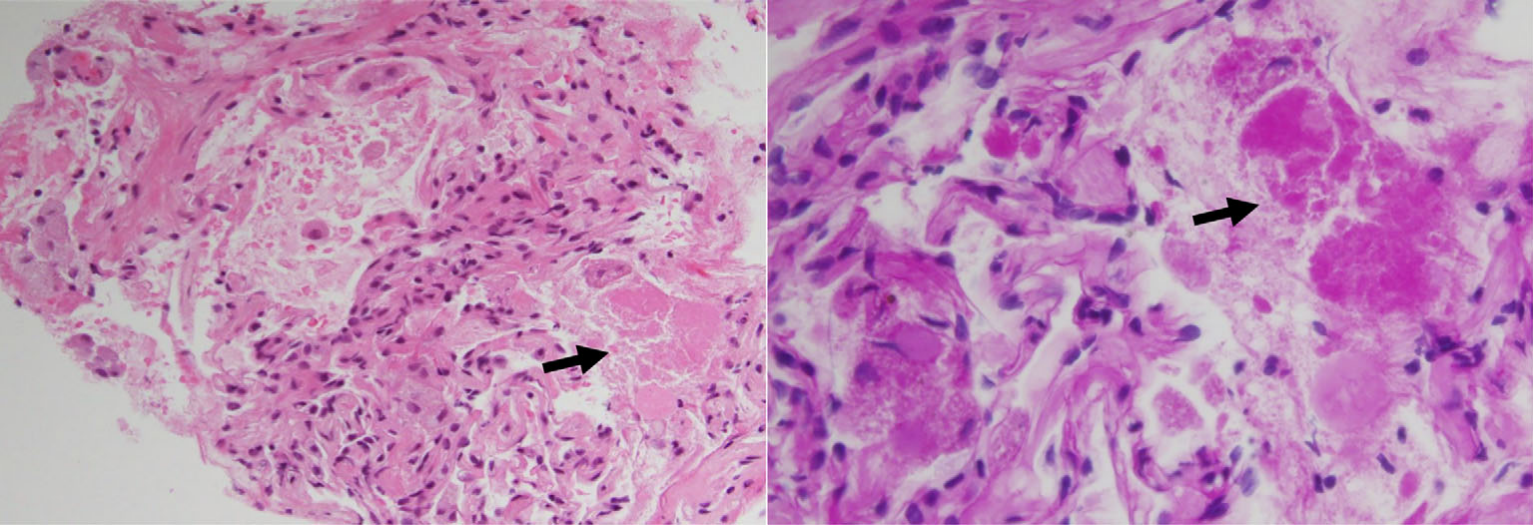
Transbronchial biopsy specimens. (A) Haematoxylin and eosin stain (200×). Black arrow indicates alveolar lung parenchyma with eosinophilic granular material filling alveolar space. (B) Haematoxylin and eosin stain (400×). Black arrow indicates PASD (periodic acid‐Schiff positive and resistant to digestion by diastase)‐positive material filling the alveolar space.

Initial blood test showed normal full blood count, urea and electrolytes, liver function test, and C‐reactive protein. High‐resolution computed tomography (HRCT) of the chest revealed bilateral widespread ground‐glass opacification with superimposed septal thickening giving a crazy‐paving appearance (Fig. 1). Resting echocardiogram and spirometry were normal. Carbon monoxide transfer factor was moderately reduced (52% predicted). Bronchoscopy was performed. Bronchoalveolar lavage (BAL) fluid showed a cloudy appearance with microscopic examination revealing dense proteinaceous material and scattered macrophages. The proteinaceous material was periodic acid‐Schiff positive and resistant to digestion by diastase (PASD positive). Transbronchial biopsies demonstrated granular eosinophilic material filling the alveolar spaces on haematoxylin and eosin staining. The eosinophilic material was also PASD positive (Fig. 2). GM‐CSF autoantibody levels were positive at 0.52 optical density units (normal <0.23). On the basis of these findings, the patient was diagnosed with autoimmune PAP.

Sulfasalazine was discontinued and the patient underwent bilateral whole lung lavage at a specialist centre. At the time of discharge, the patient could ambulate 250–300 m without oxygen desaturation on a 6‐min walk test. Over the next 13 months, the patient's exercise capacity had returned to normal. Carbon monoxide transfer factor improved to 62% predicted and chest HRCT showed near‐complete resolution of the initial changes.

In April 2019, 14 months after the diagnosis of PAP, the patient was found to be pancytopenic on full blood count performed to evaluate new breathlessness (haemoglobin 98 g/L, white cell count 2.7 × 10^9^/L, and platelet 120 × 10^9^/L). The blood film also showed 7% blast cells. Physical examination was normal and an urgent bone marrow biopsy confirmed the diagnosis of MDS. The patient was commenced on azacitidine and has remained stable to date with adequate control of MDS and no recurrence of PAP on chest HRCT.

## Discussion

PAP is a rare disorder first described by Rosen et al. in 1958 [1]. PAP is characterized by accumulation of amorphous, PASD‐positive lipoproteinaceous material within the pulmonary alveoli as a consequence of poor surfactant clearance and altered surfactant homeostasis in the lung due to impaired alveolar macrophage function [2].

Autoimmune PAP accounts for 90–95% of adult PAP and is defined by the presence of GM‐CSF autoantibodies. These antibodies neutralize the biologic activity of circulating GM‐CSF, disrupting the normal development and functioning of alveolar macrophages. Secondary PAP makes up 5–10% of PAP cases and is not associated with GM‐CSF autoantibodies. Alveolar macrophage dysfunction in secondary PAP is due to systemic conditions or toxic exposures, with haematological malignancies being the most common [3]. Congenital and unclassified PAP account for <1% of cases. Overall, autoimmune diseases have only been identified in <2% of PAP cases [4, 5]. The sensitivity and specificity of GM‐CSF autoantibodies approach 100%, making it the most useful diagnostic test in distinguishing autoimmune from secondary and unclassified PAP [6].

Key investigative tests include chest HRCT and bronchoscopy. The classic crazy‐paving pattern, which refers to the appearance of ground‐glass opacities with superimposed interlobular and intralobular septal thickening seen on chest HRCT, is present in up to 83% of patients with autoimmune PAP [7, 8]. Crazy paving is unusual in secondary PAP, with diffuse ground‐glass opacities being the most common HRCT finding [3].

Characteristic BAL findings of PAP include an opaque or milky white lavage return, and cytopathology showing engorgement of alveolar macrophages with PASD‐positive material on a background of flocculent PASD‐positive proteinaceous material [4, 9]. Addition of transbronchial lung biopsies increases diagnostic yield to ~85%, with the remaining 10–20% of patients requiring surgical biopsy for diagnosis [5, 10].

In our case, autoimmune PAP was considered to be the diagnosis based on the classic crazy‐paving pattern seen on HRCT, characteristic BAL, and transbronchial biopsy findings, as well as the presence of GM‐CSF autoantibodies. The development of MDS 14 months later was unexpected for two reasons. First, MDS, while commonly associated with secondary PAP, has not been reported to occur in autoimmune PAP. Second, MDS, if related to PAP, usually precedes the diagnosis of PAP and not after.

Two cases of autoimmune PAP associated with haematological malignancies have been described by Yoshimura et al. and Imoto et al. in 2014 and 2017, respectively [11, 12]. The first case involved a patient with chronic myeloid leukaemia who was diagnosed with PAP after commencing treatment with Abelson‐tyrosine kinase inhibitor. While GM‐CSF autoantibodies were detected, it was speculated that tyrosine kinase inhibitor‐induced mechanisms were involved. The second case reported PAP diagnosed at the same time as a myeloproliferative disorder. Although the authors regarded their case as one of autoimmune PAP (on the basis of positive GM‐CSF autoantibodies) with independent myeloproliferative disorder, given the simultaneous occurrence of both conditions, there remains a possibility that the onset of PAP was secondary to the haematological malignancy. As far as we know, our case is the first to describe autoimmune PAP associated with and preceding MDS, without any other identifiable precipitant.

It is worth mentioning that unlike other autoimmune connective tissue diseases, the association between PMR and haematological malignancies remains unclear with several studies reporting contradictory findings [13, 14]. A recent systematic review found weak evidence of an association between PMR and possibly cancer particularly in the first six months following PMR diagnosis but not after this time period, raising the possibility of initial misdiagnosis [15]. Lymphoma, myeloma, and myeloid disorders were the most commonly reported haematological malignancies. Importantly, these findings should be interpreted with caution due to the poor quality of majority of the studies analysed.

Lung involvement in PMR is rare and has not been well studied in comparison to other autoimmune connective tissue diseases. Ground‐glass opacity was reported to be the most common finding on thin slice chest CT in a small retrospective series, although its natural history remains unclear [16]. Only one case of comorbid PMR have been found in large studies of patients with PAP [4, 5]. On the basis of our current understanding of PMR, we believe it is unlikely to be implicated in the development of PAP or MDS in this case, however, this cannot be definitely excluded.

The distinction between autoimmune and secondary PAP has important therapeutic and prognostic implications. Whole lung lavage, which our patient underwent, is considered the standard of care in individuals who are symptomatic, hypoxaemic, or have moderate to severely reduced carbon monoxide transfer factor, with retrospective data suggesting improvement in symptoms and increased survival [17]. Inhalational GM‐CSF and rituximab may also be effective for autoimmune PAP [18, 19]. This was not considered in our case given the rapid improvement in symptoms after whole lung lavage. Conversely, the mainstay of treatment in secondary PAP is directed towards the underlying cause, and in the case of haematological malignancies, include chemotherapy and haematopoietic stem cell transplantation.

Although PAP secondary to haematological malignancies may improve with control of the underlying disease, its overall prognosis tends to be poor, with a median survival of 14–16 months and two‐year survival rates of ~46% [3]. In contrast, five‐year survival rates for individuals with autoimmune PAP have been reported to be as high as 75–100% [4, 5].

In summary, we present the first case of autoimmune PAP associated with and preceding MDS. We raise awareness of the need to closely follow‐up, and screen patients with autoimmune PAP for haematological malignancies as these may develop up to 14 months later. We also highlight the importance of distinguishing autoimmune from secondary PAP due to the difference in treatment options and overall prognosis.

### Disclosure Statement

Appropriate written informed consent was obtained for publication of this case report and accompanying images.
